# Framework for In Silico Toxicity Screening of Novel Odorants

**DOI:** 10.3390/toxics13100902

**Published:** 2025-10-21

**Authors:** Isaac Mohar, Brad C. Hansen, Destiny M. Hollowed, Joel D. Mainland

**Affiliations:** 1 Gradient, Seattle, WA 98104, USA; 2 School of Public Health, University of Washington, Seattle, WA 98195, USA; bhansen3@uw.edu; 3 Independent Researcher; destinyhollowed@gmail.com; 4 Monell Chemical Senses Center, Philadelphia, PA 19104, USA; jmainland@monell.org; 5 Department of Neuroscience, University of Pennsylvania, Philadelphia, PA 19104, USA

**Keywords:** in silico, inhalation, concentration, exposure, toxicity, prediction

## Abstract

Toxicological risk assessment of chemicals without experimental toxicity data often relies on in silico predictions. However, models designed to predict inhalation toxicity associated with exposure to volatile chemicals in solution are unavailable. The aim of this research was to develop an approach to estimate toxicology-based maximum solution concentrations for novel odorants using in silico structure-based predictions. The decision trees were adapted from established open-source models for assessing mutagenicity (rule-based, ISS in vitro mutagenicity decision tree) and systemic toxicity (revised Cramer decision tree). These were implemented using Toxtree (v3.1.0), a freely available program. Thresholds of toxicologic concern (TTC) were then assigned based on the predicted hazard classification. We then used predicted vapor pressure derived from MPBPWIN™ using US EPA EPI Suite to calculate a solution concentration where inhalation exposure to a defined headspace volume would not exceed the TTC. The approach was evaluated using a published dataset of 143 chemicals with repeat exposure inhalation toxicity data, yielding health-protective predictions for 98.6% of the test set. This demonstrates that the proposed in silico approach enables the estimation of safe toxicology-based maximum solution concentrations for chemicals using open-source models and software.

## 1. Introduction

In silico programs are widely used as predictive tools to screen chemicals lacking experimental toxicity data, helping toxicologists and risk assessors identify and prioritize substances of potential concern. Although in silico prediction tools are widely applied in chemical screening and risk management for classical toxicology endpoints [1], to our knowledge, there are no existing transparent and widely available in silico structure-activity models for predicting inhalation toxicity for volatile chemicals in solution. The aim of this research was to develop an intuitive and user-friendly approach to estimate toxicology-based maximum solution concentrations for novel odorants using in silico structure-based predictions.

The approach was developed to support a psychophysical study of novel odorants, in which human volunteers sniffed the headspace of a solution in a vial [2]. The novel odorants evaluated in the study by Lee et al. [2] were designed and selected to represent key molecular and perceptual features of odorants and to validate model predictions against the odor description reported by human volunteers. Predicting odor perception from molecular structure remains a key challenge in olfaction, and validating perceptual models requires testing novel odorants that often lack safety data. The inhalation in silico framework presented in this paper enabled a preliminary toxicological assessment of candidate odorant structures and provided estimates of safe maximum solution concentrations.

Importantly, this in silico framework was developed and assessed for safety predictions independently of the novel odorant olfaction study with the objective of providing a practical and transparent tool for screening inhalation toxicity risks of chemicals in solution. The method combines freely available and established in silico methods to predict mutagenicity hazard, systemic toxicity hazard, and vapor pressure and adapted these to inhalation exposure in a defined headspace.

The expert rule-based in vitro mutagenicity (Ames test) decision tree by Istituto Superiore di Sanita (ISS)/Benigni and Bossa was used to predict mutagenicity hazard [3,4]. The ISS decision tree is commonly used for mutagenicity predictions of data poor chemicals and, in addition to Toxtree (version 3.1.0) [5], is a bacterial mutagenicity prediction module in VEGA QSAR (version 1.1.5) and OECD QSAR Toolbox (version 4.5) programs [6,7]. The ISS decision tree within Toxtree has comparable sensitivity and specificity to other rule-based mutagenicity prediction models [8,9].

The revised Cramer tree was used to predict systemic toxicity hazard for potential repeated exposure. Based on the original decision tree developed by Cramer and Ford [10], the revised Cramer tree in Toxtree (version 3.1.0) was developed as a collaboration between IdeaConsult, Ltd., the International Organization of the Flavor Industry, and the Flavor and Extract Manufacturers Association [11]. The Cramer tree is widely used for in silico systemic toxicity prediction of data poor chemicals, including indirect food additives, pesticides, flavoring, cosmetic ingredients, and fragrances across multiple exposure routes, including oral, dermal, and inhalation [10,12,13,14,15]. The Cramer tree is also used in the safety assessment of extractable compounds from medical devices [16].

Lastly, vapor pressure was predicted using the MPBPWIN™ v.1.43 model available through the EPI Suite Program (version 4.1), published and peer reviewed by the US Environmental Protection Agency [17].

This paper provides a thorough description and example application of the silico framework for deriving toxicology-based maximum solution concentrations using open-source models and software.

## 2. Materials and Methods

### 2.1. Model Description

The in silico inhalation toxicity screening method was based on two well-established and accepted in silico methods for mutagenicity and chronic toxicity hazards and was adapted to inhalation exposure of a defined headspace under standard atmospheric temperature and pressure.

**Figure 1:**
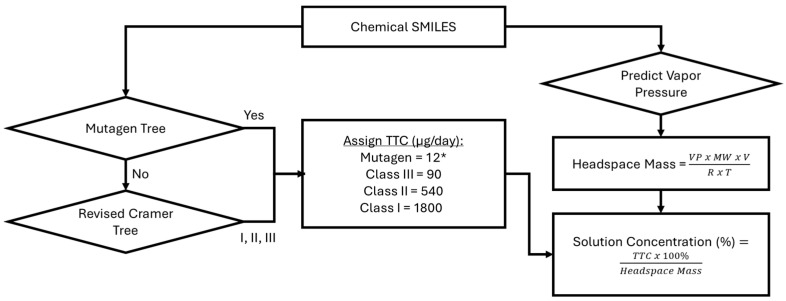
Overview of In Silico Prediction of Inhalation Toxicity Hazard and Solution Concentration. MW = Molecular Weight; R = Ideal Gas Constant; SMILES = Simplified Molecular Input Line Entry System; T = Temperature; TTC = Threshold of Toxicological Concern; V = Headspace Volume; VP = Vapor Pressure. * For the mutagenic TTC, a value of 12 μg/day was adopted based on the acute exposure scenario (less than 30 days) at an excess cancer risk of 1:1,000,000 [18].

As illustrated in Figure 1, the revised Cramer decision tree [11] and rule-based bacterial mutagenicity Ames tree [3,4] were adapted to the context of inhaled chemicals. Both models are validated and accepted in silico methods for identifying potentially toxic chemicals and/or mutagenic compounds, respectively, and are built-in decision trees in the open-source in silico program Toxtree (v.3.1) [5].

The composite inhalation toxicity prediction model shown in Figure 1 is a series of toxicity predictor modules—mutagenicity (Ames) tree and revised Cramer tree—with a parallel physical chemistry module. The input is Simplified Molecular Input Line Entry System (SMILES) notation, which can be generated from chemical structures.

From this series of trees, the highest (i.e., most toxic) hazard prediction is assigned to each chemical as either mutagen, Cramer Class III, Class II, or Class I (highest to lowest hazard).

From the hazard predictions (i.e., mutagen, Class I, Class II, or Class III), a Threshold of Toxicological Concern (TTC) was assigned to each queried chemical SMILES, as summarized in Table 1. For the mutagenic TTC, a value of 12 μg/day was adopted based on the acute exposure scenario (less than 30 days) at an excess cancer risk of 1:1,000,000 [18]. For the non-mutagenic TTC values, Kroes TTCs were applied based on the predicted Cramer classification [12,13]. Kroes TTC values were developed for risk assessment of dietary exposures but are used by toxicologists for screening of data-poor chemicals in fragrance and medical device safety assessment [15,16].

The toxicity predictions can be used as a stand-alone screening method and to provide a health-protective exposure limit. However, the practical utility of these values for an inhaled chemical required additional considerations of airborne concentration and duration (or fixed volume) of exposure. As an extension of the predicted exposure limits (in mass per day), a physical chemistry module was integrated into the method using the predicted vapor pressure of the chemical.

The vapor pressure values for query compounds were calculated using the MPBPWIN™ v.1.43 model available through the EPI Suite Program, published and peer reviewed by the US Environmental Protection Agency [17]. This model uses chemical inputs in the SMILES notation and provides a model prediction value in mm Hg at 25 °C. Notably, the EPI Suite program has a linked dataset and will provide an experimental value with a citation if available for the chemical of interest.

Assuming ideal gas behavior (i.e., PV = nRT) under fixed headspace volume (V) and temperature (T), the mass of the chemical in the headspace will depend on the chemical-specific vapor pressure (VP) and molecular weight (MW). Based on the predicted (or experimental) vapor pressure, the mass of the chemical derived from a neat (i.g., 100%) solution in a defined headspace was calculated as follows:

Headspace Mass = (VP × MW × V) ÷ (R × T).(1)

As illustrated in Figure 1, following assignment of hazard and an exposure limit (TTC) to a chemical, an allowable concentration in solution was derived by dividing the TTC by the estimated mass in the headspace volume for a neat solution (i.e., exposure) as follows:

Concentration (% *w*/*w*) = (TTC μg/day × 100%) ÷ (Exposure μg/day). (2)

By this approach, the calculation provides an adjusted chemical concentration where inhalation of the headspace mass would not exceed a hazard-based TTC and therefore would be theoretically safe.

This approach assumes ideal gas behavior and that the mass of the chemical in the headspace is at equilibrium with the solution and will be directly proportional to the concentration in solution and total headspace volume. Thus, for chemicals that are relatively non-volatile, regardless of the assigned TTC, very little chemical would be in a headspace and therefore the allowable concentration in a solution is likely to be high (i.e., ~100% for a relatively small headspace). In contrast, highly volatile chemicals may readily enter the headspace, and exposure may therefore need to be limited by reducing the allowable solution concentration. Importantly, the approach would adjust the solution concentration inversely proportional to the headspace volume. For example, a 10-fold increase in headspace volume (i.e., inhaled volume) would decrease the solution concentration by 10-fold.

### 2.2. Model Safety Testing

The approach was tested for predicting adequately safe exposure limits using a published dataset of 143 chemicals with repeat dose inhalation toxicity data [19]. An allowable solution concentration was derived for each chemical using the above approach. For chemicals with allowable concentrations less than 100%, multiple solvents were considered (100% propylene glycol, 100% water, and 50% ethanol in water), and the partial pressure of the query chemical in the headspace was derived based on the solvent-specific mole fraction multiplied by the predicted (or experimental) vapor pressure, as follows:

Partial Pressure (mmHg) = [(Chemical mol/L) ÷ (Chemical mol/L + Solvent mol/L)] × VP (mmHg)(3)

where the adjusted headspace mass was then calculated using the partial pressure in Equation (1).

The no observed adverse effect concentration (NOAEC) values reported by Shin et al. [19] were used to derive a no observed adverse effect level (NOAEL) in units of μg per day using the ICH default breathing rate of 0.2 L/min for rats [20] and 360 min per day exposure, which are the standard exposure duration and test species for industrial inhalation toxicity studies conducted in accordance with OECD guideline [21]. From this, a safety margin was derived as a ratio of the experimental NOAEL and adjusted headspace exposure. A value greater than or equal to 1 would support concluding that the approach predicted a concentration that would not pose a health risk.

## 3. Results

### 3.1. Model Safety Testing

**Figure 2:**
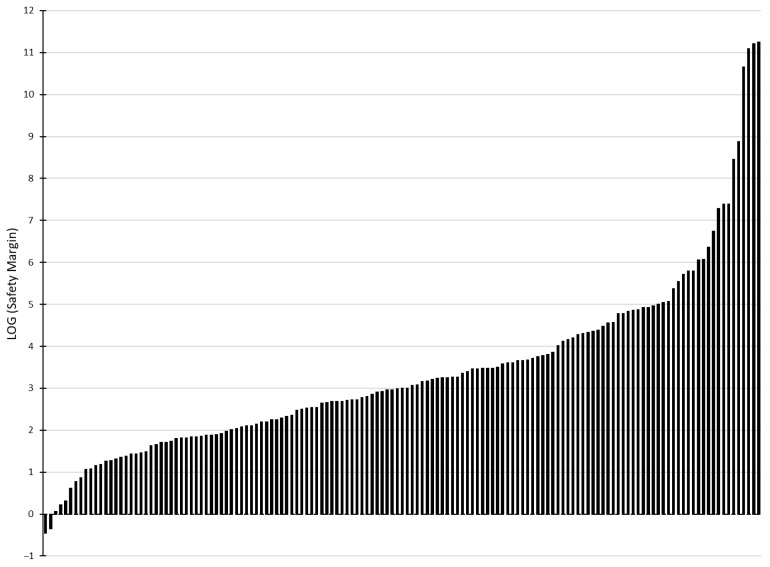
Safety Margins of Experimental Test Dataset. A comparison of the experimentally derived NOAELs from Shin et al. [19] to the predicted chemical mass in the headspace using the described in silico framework. A Log(safety margin) greater than 0 indicates that the model-derived exposure limit is less than the experimental NOAEL. As illustrated in this figure, two of the 143 chemicals had a NOAEL below the predicted maximum exposure based on the derived solution concentration in water.

Testing the safety of the model approach and predictions against a dataset of 143 chemicals with repeated-dose inhalation toxicity data [19] demonstrated that the proposed approach provides health-protective solution concentration limits. Using water as a solvent, safety margins were greater than one for 98.6% of chemicals and greater than ten for 94.4%. Only 2 of the 143 experimentally derived NOAELs fell below the predicted headspace mass expected at equilibrium in a 100 mL headspace volume (Table 2, Figure 2, Supplemental Data).

### 3.2. Solvent Effect

**Figure A1:**
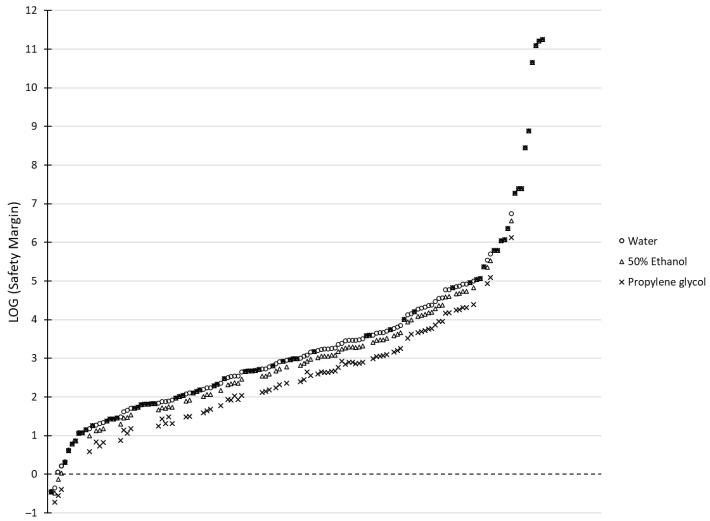
Safety Margins of Experimental Test Dataset. A comparison of the experimentally derived NOAELs from Shin et al. [19] to the predicted chemical mass in the headspace using the described in silico framework with water, 50% ethanol, or propylene glycol as solvents. A Log(safety margin) greater than 0 indicates that the model-derived exposure limit is less than the experimental NOAEL.

The influence of solvent on model performance was minimal. For 50% ethanol and 100% propylene glycol, out of the 143 validation chemicals, the derived safety margins were less than one for 3 and 4, respectively, while the number of chemicals with safety margins less than ten were 9 and 13, respectively (Table 2, Figure A1, Supplemental Data).

### 3.3. Case Example, Rose Oxide

Rose oxide is provided as an example case study of the workflow as summarized in Table 3. The predicted exposure was 546 μg derived from a predicted vapor pressure of 0.657 mmHg for a 100 mL headspace volume at 25 °C (equivalent to 298 K), as follows:

[0.657 mmHg × 0.001316 atm/mmHg × 154.25 g/mol × 0.1 L × 1 × 10^6^ µg/g] ÷ [0.082 (L-atm)/(K-mol) × 298 K] = 546 μg (4)

Assuming once-daily exposure, this corresponds to daily exposure of 546 μg/day.

Integrating the toxicology prediction and assigned TTC of 540 μg/day with the physicochemical prediction and a calculated headspace mass of 546 μg (i.e., 546 μg/day exposure), the in silico framework predicted a maximum safe solution concentration in water as follows:

[540 μg/day × 100%] ÷ [546 μg/day] = 98.9%.(5)

## 4. Discussion

In this study, toxicology-based maximum recommended solution concentrations for odorant chemicals were derived using an in silico approach based solely on chemical structure. This framework was developed to support a psychophysical study of novel odorants in human volunteers.

The adaptation of the revised Cramer decision tree and bacterial mutagenicity (Ames) decision trees with standard TTC values is a practical, transparent, and toxicologically validated approach to develop a health-protective in silico screening model. As noted above, these models have been thoroughly validated across broad chemical classes and are used in numerous applications. Incorporating vapor pressure into the framework addressed inhalation exposure in a straightforward manner: relatively non-volatile chemicals contribute little to the headspace and are therefore often permissible at concentrations approaching 100%, while highly volatile chemicals may require reduced solution concentrations to limit exposure.

The repeat-dose inhalation toxicity data referenced in this paper and used to test the safety of our model predictions were collected from the publication by Shin et al. [19]. The paper by Shin et al. [19] provides a conceptual description and reports the statistical performance of a quantitative structure activity relationship (QSAR) random forest model that predicts a no observed adverse effect concentration (NOAEC). While seemingly ideal for our desired application, the paper lacks the methodological specifics to allow for the approach to be used by others. Furthermore, while predicting a NOAEC is useful for certain applications, the NOAEC does not itself provide a limit for a safe daily exposure (i.e., μg/day).

Nearly all NOAELs in the validation dataset were derived from repeated-dose inhalation studies in animals (6 h/day for up to 90 days). With water as the solvent, the lowest safety margins were for cyanuric chloride (CAS No. 108-77-0) and acetyl acetate (CAS No. 108-24-7). The NOAECs for both chemicals were based on local irritation of the respiratory system with chronic exposure [22,23], which is an effect that would not be expected to occur in an acute exposure scenario (such as that for the novel odorant study) but may be relevant to longer term repeated exposure.

Although solvent did not alter the predicted maximum solution concentration for a chemical, it did affect potential exposure by influencing mole fraction, partial pressure, and headspace mass. As a result, safety margins varied by solvent (Table 2).

The proposed method is health-protective in the context of acute exposure for two reasons. First, TTCs are derived to be protective for chronic, repeated exposures. For example, the mutagenicity TTC of 12 μg/day, exposure could occur daily for up to 30 days at a risk level of at least 1:1,000,000 [18]. In the case of the Kroes TTC values, daily lifelong exposure could occur [12]. These thresholds are therefore highly conservative for single or brief exposures, as in the novel odorant study. Second, the model identifies both potentially hazardous chemicals and highly volatile chemicals, minimizing the likelihood of exposures exceeding toxicological limits.

The conservativeness of the TTC values is reflected in the safety margins derived against the experimental data, where the geometric mean safety margin was greater than 1000 across all solvents (Table 2). It is important to emphasize that TTC values are most appropriately used in the toxicological risk assessment of chemicals that lack relevant toxicity data, as was the case for the theoretical novel odorants for which this risk assessment approach was developed.

There are limitations associated with this approach. The TTC values were derived from oral toxicity data and extrapolated to inhalation exposure. The inhalation exposure route could potentially allow for greater bioavailability relative to the oral route. However, for acute exposure scenarios, the chronic oral toxicity TTC values can be expected to be health protective against systemic toxicity. For example, the TTC values used in the framework may be applied to the toxicological risk assessment of permanently implanted medical devices without modification [16] and appear to be robust safety thresholds for fragrances [15].

Certain chemical classes, such as organophosphates, dioxins, alkyl azoxy compounds, N-nitrosoamines, and aflatoxin-like chemicals, may have lower safe exposure values than those predicted by the method and should therefore be excluded from analysis by this method [16,18].

A notable limitation is that the approach may not identify asthmagens, and TTC values may be inadequate to guard against elicitation of an asthmatic reaction. Similarly, this approach may not be able to completely safeguard against irritation of the nose, eyes, or lungs. However, in an acute exposure scenario, mild irritation that would occur can be expected to be temporary, and to some degree, the exposure limit for systemic toxicity can be expected to guard against severe irritation. There are, however, toxic gases, such as chlorine gas, where damage to the respiratory tract may worsen over time. Nevertheless, the above framework would set a limit of 0.005% for chlorine (Cl2) in water resulting in a maximum inhaled dose of 23 μg from a 0.1 L headspace. This predicted exposure is less than that which would result from inhalation of chlorine gas for 1 min at the US EPA acute exposure guideline level (AEGL)-1 value of 1.5 mg/m3, which is a level that would not be expected to cause meaningful effects [24] and provides reassurance that the proposed framework would be robust to relatively potent irritants.

Importantly, TTC values used here are based on repeated daily exposures and are therefore expected to be conservative for brief exposures such as those in psychophysical testing of novel odorants. If the in silico method is unable to assign predictions for compounds of interest or chemical exposures differ significantly from the described scenario, additional considerations of the method or the exposure limits would be warranted.

## 5. Conclusions

This in silico inhalation toxicity screening approach provides a transparent, practical, and health-protective method to support a psychophysical study of novel odorants. Although best applied as a screening tool and subject to certain limitations, safety testing against existing data indicates it provides conservative health-protective estimates of maximum solution concentrations. The use of freely available open-source software enhances transparency and allows for further refinement and application of the method.

## Figures and Tables

**Table 1:** Summary of Hazard Prediction and Associated TTC Value.

Hazard Prediction	TTC (μg/day)
Mutagen	12 ^1^
Cramer Class III	90
Cramer Class II	540
Cramer Class I	1800

TTC = Threshold of Toxicological Concern. ^1^ As described in the ICH M7(R2) guidance, for less than 30 days exposure, a less-than-lifetime mutagenic TTC of 120 μg/day would result in a theoretical excess risk of 1 × 10^−5^ to 1 × 10^−6^; this value was adjusted to a TTC value of 12 μg/day at a theoretical excess risk of at most 1 × 10^−6^ [18].

**Table 2:** Summary of Model Performance by Solvent.

Safety Margin Statistics	Solvent		
	Propylene Glycol	Water	Ethanol/Water (50:50)
Geometric Mean (range)	1124 (0.19–1.78 × 10^11^)	2358 (0.35–1.78 × 10^11^)	1878 (0.33–1.78 × 10^11^)
<1 (No. chemicals)	4	2	3
<10 (No. chemicals)	13	8	9

**Table 3:** Summary of Rose Oxide Assessment.

Parameter	Value
Chemical Description	
Name	4-methyl-2-(2-methylprop-1-enyl)oxane (rose oxide)
CAS No.	16409-43-1
Molecular Formula	C^10^H^18^O
SMILES	CC1CCOC(C1)C=C(C)C
Toxicology Predictions	
Mutagenicity	No alerts
Cramer Classification	Intermediate (Class II)
Assigned TTC	540 μg/day
Physicochemical Predictions	
Molecular Weight	154.25 g/mol
Vapor Pressure (predicted at 25 °C)	0.657 mmHg
Headspace mass (V = 0.1 L)	546 μg

## Data Availability

The original contributions presented in this study are included in the article/Supplementary Materials. Further inquiries can be directed to the corresponding author.

## Supplementary Materials

The following supporting information can be downloaded at: https://www.mdpi.com/article/10.3390/toxics13100902/s1. Excel File: Mohar et al-Source-Data.
